# Knowledge and Prevalence of Latent Tuberculosis Infection: A Feasibility and Pilot Study in a Primary Healthcare Setting in Rural Eastern Cape, South Africa

**DOI:** 10.3390/ijerph22030320

**Published:** 2025-02-21

**Authors:** Cebo Magwaza, Oluwakemi Laguda-Akingba, Teke Apalata, Lindiwe Modest Faye

**Affiliations:** 1 Department of Laboratory Medicine and Pathology, Walter Sisulu University, Private Bag X5117, Mthatha 5099, South Africa; magwazacebo13@gmail.com (C.M.); olaguda-akingba@wsu.ac.za (O.L.-A.); tapalata@wsu.ac.za (T.A.); 2 Virology Department, National Health Laboratory Service, Port Elizabeth 6001, South Africa

**Keywords:** latent tuberculosis infection, knowledge, prevalence, comorbidities, gender, age, education

## Abstract

Latent tuberculosis infection (LTBI) remains a significant global health concern, particularly in regions with high tuberculosis (TB) prevalence, such as South Africa. This pilot study aimed to evaluate the prevalence of LTBI and assess patient knowledge about the condition in a primary healthcare clinic in rural Eastern Cape, South Africa. A cross-sectional design was used, and convenience sampling recruited outpatients aged 18 years and older with no prior history of TB. Blood samples were analyzed using the QuantiFERON-TB Gold assay to determine LTBI status, and a survey assessed patient knowledge of LTBI. Strong positive correlations were observed between what patients understand by the term LTBI and how LTBI differs from TB (0.70), what patients understand by the term LTBI and the risk factors for developing LTBI (0.70), how LTBI differs from TB and the risk factors for developing LTBI (0.78), and how LTBI differs from TB and the recommended treatments for LTBI (0.79), indicating overlap in understanding. In contrast, there were negative correlations between if patients had ever heard of latent LTBI before and their understanding of the term LTBI (−0.25), the risk factors for developing LTBI (−0.22), LTBI progressing to active TB (−0.27), and the recommended treatments for LTBI (−0.27). This divergence points to different dimensions of patient knowledge and awareness. Age, gender, occupation, comorbidities, and HIV status showed varying LTBI positivity trends. Among younger patients aged 20–29, 15.4% tested positive, while the 30–39 group showed a nearly equal split between positive (48.1%) and negative cases. A higher positivity rate was seen in females (39.1%) compared to males (31.6%). Unemployed individuals had higher positivity rates, suggesting socioeconomic factors’ influence. Comorbidities, especially hypertension, diabetes, and asthma, correlated with higher LTBI positivity among females, but this was less evident in males. HIV-positive patients had a higher LTBI-negative rate compared to HIV-negative patients. A logistic regression model (accuracy 70%) identified demographic and health factors predicting LTBI outcomes, with comorbidities, particularly hypertension and diabetes, significantly increasing the likelihood of LTBI positivity. These findings suggest that demographic and health factors, including age, gender, occupation, comorbidities, and HIV status, may predict LTBI positivity.

## 1. Introduction

Latent tuberculosis infection (LTBI) is a state where individuals are infected with *Mycobacterium tuberculosis* but do not exhibit symptoms of active tuberculosis (TB). The global burden of LTBI is substantial, with an estimated one-quarter of the world’s population affected, particularly in regions with high TB incidence, such as South Africa [1]. Detecting LTBI early is crucial for the World Health Organization’s (WHO) End Tuberculosis Strategy [2]. *Mycobacterium tuberculosis* (*Mtb*) poses a significant threat to public health worldwide, with 10 million people developing TB and 1.6 million succumbing to it annually. *Mtb* has shared a long evolutionary history with humans, exerting its influence over millennia [3]. One key factor contributing to its effectiveness as a pathogen lies in its capacity to reside within its host without causing symptoms, entering a dormant state known as latency [3]. Later, in a minority of cases, it can progress to active TB after months or even years of dormancy. The likelihood of LTBI reactivating into TB during a person’s lifetime varies depending on their age at the time of infection and the presence of any additional medical conditions associated with TB progression [3]. The majority of individuals who are exposed to *Mtb* develop LTBI and remain susceptible to progressing to active TB [4]. While most TB patients have LTBI, only a small percentage (about 5–15%), develop symptomatic illness. However, individuals with active infection face the risk of reactivation even after completing treatment. Therefore, diagnosing LTBI is critical in TB management to prevent its progression to active TB [5]. Early detection of LTBI, particularly in high-prevalence settings, aligns with THE WHO’s End TB Strategy, which aims to reduce TB incidence by 90% and TB-related deaths by 95% by 2035.

Screening for LTBI is crucial in global TB control, particularly in high-risk and high-prevalence settings, as it helps identify asymptomatic individuals with latent *Mycobacterium tuberculosis* infections. LTBI screening, followed by treatment, prevents progression to active TB, reducing transmission, morbidity, and mortality. This review synthesizes the evidence on LTBI screening efficacy across populations. Studies indicate that LTBI screening in high-risk groups, such as healthcare workers, migrants, and those with comorbidities, substantially reduces TB incidence. For instance, screening healthcare workers in high-prevalence regions lower disease progression risk [6]. Migrant screening programs in low-incidence countries like the UK and the US show up to a 60% reduction in TB incidence within five years of entry screening [7,8]. This approach is also cost-effective, as early LTBI detection is generally cheaper than treating active TB cases [7,8]. LTBI screening in human immunodeficiency virus (HIV) patients, who are at high risk for reactivation, reduces TB incidence by nearly 50% with isoniazid preventive therapy (IPT) [9]. In sub-Saharan Africa, integrating LTBI screening with HIV care improves TB control [10]. Community-based LTBI programs also succeed in high-incidence areas. In China, a pilot program reduced local TB incidence by 20% in five years [11]. Similar success is reported in rural South Africa [12]. LTBI screening is also effective among household contacts of active TB cases. Screening followed by treatment reduces secondary TB by over 70%, which is crucial for protecting vulnerable groups [13].

Tuberculosis remains a significant contributor to global mortality from infectious diseases despite the availability of effective treatment and prevention methods for both active and latent TB [14,15,16,17]. This means that a large proportion of TB-related deaths could be avoided. With approximately one-quarter of the world’s population harboring LTBI, it is a reservoir for initiating new TB cases [18]. Many of the world’s population carry the *Mtb* infection, with the majority displaying no tuberculosis disease symptoms and posing no infectious risk [19]. Nonetheless, these individuals are still vulnerable to developing active TB disease and potentially becoming contagious [20].

The WHO recently released a new report on tuberculosis, revealing that around 8.2 million people were newly diagnosed with TB in 2023, the highest figure recorded since the WHO began tracking global TB cases in 1995 [21]. This marks a significant increase from the 7.5 million cases reported in 2022, reestablishing TB as the leading cause of death from infectious disease in 2023, overtaking COVID-19. The WHO’s Global Tuberculosis Report 2024 outlines mixed progress in the global fight against TB, noting persistent challenges like substantial underfunding. Although TB-related deaths decreased from 1.32 million in 2022 to 1.25 million in 2023, the total number of people affected by TB rose slightly to an estimated 10.8 million in 2023 [21].

Demographic factors such as gender and socioeconomic status also play a role in LTBI prevalence across age groups. For instance, a study in England found that LTBI positivity was higher among older migrants, with rates ranging from 25% to 30% in various studies [22]. Additionally, healthcare workers, particularly those born outside the country, showed elevated LTBI rates, emphasizing the intersection of age, occupation, and migration status [23,24].

The relationship between occupational exposure and the prevalence of LTBI is well-documented, particularly among healthcare workers (HCWs) and other high-risk occupational groups. Various studies have consistently shown that individuals working in healthcare settings, especially those with direct patient contact, exhibit significantly higher rates of LTBI compared to those in other professions. Healthcare workers, particularly nurses and medical staff, are at an elevated risk for LTBI due to their frequent exposure to patients with active TB. A systematic review indicated that HCWs who have more direct contact with TB patients or prolonged exposure are significantly more likely to test positive for LTBI [25]. The findings from a study in South Africa highlighted that specific work locations, such as TB wards and outpatient departments, are associated with increased risk of both active TB disease and LTBI among healthcare personnel [26]. Another study in China found that the prevalence of LTBI was markedly higher in high-risk workplaces (37.4%) compared to low-risk environments (29.8%) [27].

In this study, our primary objective was twofold: to evaluate the feasibility of assessing LTBI knowledge and prevalence in a resource-limited setting and to collect preliminary cross-sectional data on the relationships between demographic, health-related variables, and LTBI outcomes. Combining these elements allowed us to gain valuable insights while exploring the practicalities of conducting our research. Understanding the prevalence of LTBI and the knowledge surrounding it in our study setting that is burdened with TB is crucial for effective public health strategies aimed at TB control. From a public health perspective, the findings from this study have significant implications for TB control in the rural Eastern Cape of South Africa. Our research informs targeted health education strategies and community-level interventions by highlighting factors such as low awareness of LTBI, its risk factors, and the role of demographic and occupational variables in LTBI prevalence. Furthermore, the study emphasizes the need for integrating LTBI screening and educational programs into primary healthcare services, which could reduce the progression to active TB and improve overall TB management in resource-limited settings.

## 2. Materials and Methods

This pilot study was a cross-sectional study conducted at a primary healthcare clinic in rural Eastern Cape, South Africa. The clinic was selected due to its high prevalence of TB and its central role in providing primary TB care to an underserved community. This location choice supports the study’s objective of piloting an understanding of LTBI knowledge and prevalence in areas with high TB burden and limited healthcare resources. The primary aim was to gather preliminary data on the prevalence and awareness of LTBI within a primary healthcare setting in a rural, TB-affected community. Given the study’s exploratory nature, a convenience sampling method was used to recruit patients who were visiting the clinic and who met specific criteria. A total of 88 outpatients were enrolled based on the inclusion criteria of being aged 18 or older with no prior TB infection history. Exclusion criteria included a confirmed or probable active TB diagnosis and previous anti-tuberculosis treatment. As a pilot study with a limited sample size (n = 88), all eligible outpatients meeting the criteria were included to represent a diverse demographic range. Participant demographic distribution was shaped by convenience sampling within the clinic, which may have limited the overall representation of the broader community population. Given the small sample size of participants for this pilot study, saturation was considered qualitatively rather than quantitatively. Saturation was approached as the point at which recurring themes and patterns in participant responses became consistent, with minimal to no emergence of new insights. During data analysis, we observed that this occurred around the 70th participant, as additional responses largely reiterated previously identified themes regarding LTBI knowledge, risk factors, and attitudes. However, we continued data collection for all eligible participants to ensure a comprehensive representation of the study population and maintain our quantitative assessments’ statistical validity.

Patients who consented to be study participants were surveyed regarding their knowledge of LTBI following questions in Table 1 that were ensured of content validity by basing the questions on established guidelines and prior studies on TB knowledge. After completing the questionnaire, participants underwent blood collection using Quanti FERON-TB Gold blood collection tubes to test the prevalence of LTBI. Study participants’ blood samples were tested by QFTGIT assay kits from Cellestis Limited, Melbourne, VIC, Australia, following the protocol outlined by the manufacturer [28,29]; this is a widely validated tool for detecting LTBI with high sensitivity and specificity to assess the presence of LTBI. Optical density readings were recorded using a microplate reader equipped with 450- and 620-nm filters ()Molecular Devices Corporation, San Jose, CA, USA. The Quanti FERON-TB Gold Analysis Software (Cellestis Limited) was used to analyze the results.

A comprehensive analytical framework was employed to assess LTBI prevalence and knowledge using descriptive statistics, correlation analysis, logistic regression modeling, and network analysis. Descriptive statistics summarized demographic and health-related characteristics, applying means, standard deviations, frequencies, and percentages to continuous and categorical variables. Pearson correlation analysis identified significant patterns and associations between survey responses and demographic factors, particularly in relation to LTBI knowledge and behaviors. A binary logistic regression model was developed to predict LTBI positivity, incorporating variables such as age, gender, comorbidities, and survey responses, with LTBI test results serving as the binary outcome. The LTBI test result was encoded as a binary variable (0 for negative; 1 for positive). The data were split into training and testing sets to allow for model performance evaluation, and the model was trained on the training set. Model performance was assessed using metrics such as accuracy, precision, recall, and the F1 score. Feature importance was evaluated by interpreting the model coefficients, highlighting factors strongly influencing LTBI positivity. Additionally, network analysis was employed to visualize significant correlations (|correlation| > 0.3) within the dataset, where nodes represented variables and edges depicted structural relationships. Python (version 3.8) and R (version 4.1.1) software were used for data analysis. A significance level of *p* < 0.05 was considered for all statistical analyses.

## 3. Results

**Figure 1:**
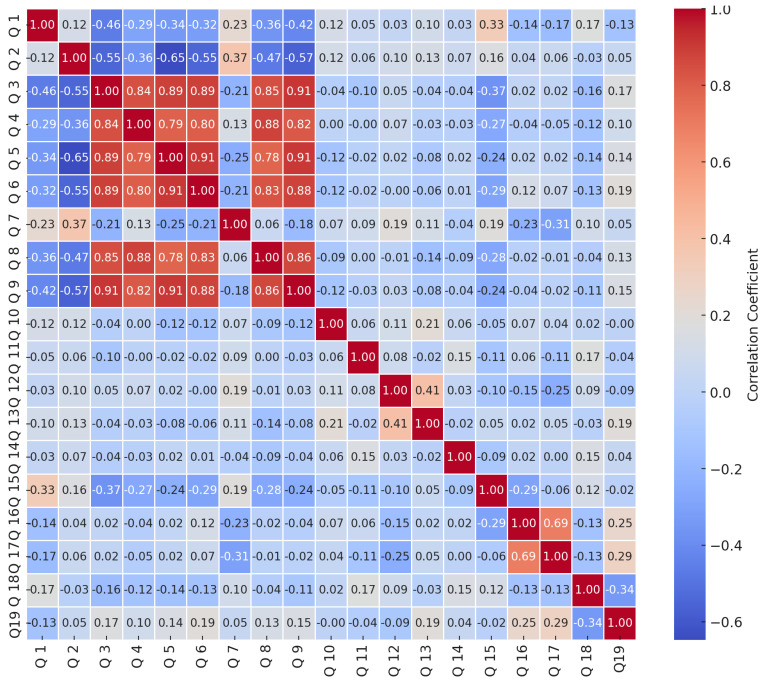
Correlation matrix for knowledge questions (Q1 to Q19).

In Figure 1, the correlation analysis of survey responses on LTBI reveals distinct patterns in respondent knowledge and attitudes. High positive correlations highlight clusters of questions that may reflect cohesive themes or shared perceptions. For instance, the strong correlation between Q3 (understanding LTBI) and Q4 (difference between LTBI and active TB) (0.84) suggests that respondents who grasp the concept of LTBI also tend to understand its distinction from active TB, indicating that these questions might address related aspects of knowledge. Similarly, Q8 (recommended LTBI treatments) and Q9 (preventive measures for LTBI), with a correlation of 0.91, appear to capture closely aligned knowledge or attitudes towards LTBI prevention and treatment. Furthermore, Q5 (risk factors of LTBI) shows strong correlations with both Q6 (consequences of untreated LTBI) (0.91) and Q3 (understanding LTBI) (0.89), suggesting a thematic grouping around LTBI risks and the implications of untreated infection, where respondents view these questions as part of a continuum of understanding regarding LTBI. In contrast, notable negative correlations reveal areas where responses diverge, potentially highlighting contrasting perspectives or knowledge gaps. For example, the strong negative correlation between Q2 (health education on LTBI/TB) and Q5 (risk factors for LTBI) (−0.65) indicates that respondents with prior health education tend to view LTBI risk factors differently, possibly due to varied levels of exposure or understanding. Additionally, a moderate negative correlation between Q7 (LTBI progression to active TB) and Q3 (understanding LTBI) (−0.21) suggests that respondents who are aware of LTBI’s potential to progress to active TB might have a differing understanding of LTBI itself, hinting at a divergence in knowledge or perspectives. Lastly, weaker negative correlations, such as between Q10 (LTBI as a public health concern) and Q3 (understanding LTBI) (−0.04), hint at subtle differences where respondents who view LTBI as a public health issue may have a slightly different perception or understanding of LTBI, indicating a potential gap between general concerns and specific knowledge. These correlation patterns underscore areas of thematic alignment as well as contrasting perspectives, offering insights into respondents’ understanding and attitudes towards various aspects of LTBI.

**Figure 2:**
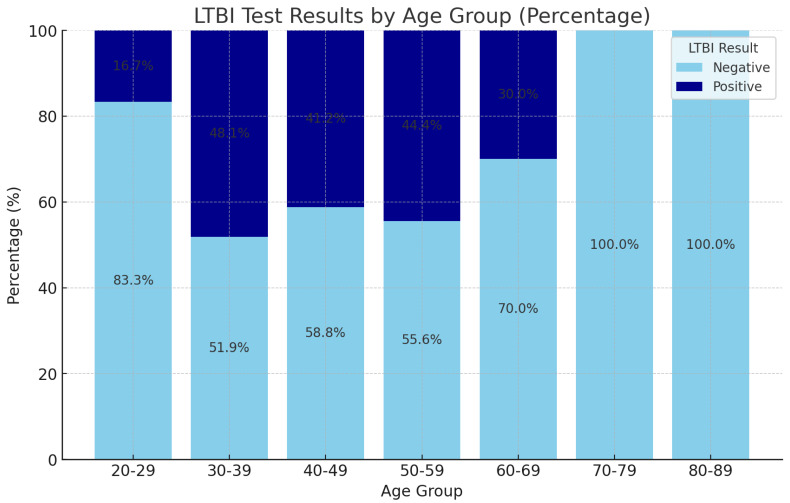
LTBI results by age group.

In Figure 2, the highest LTBI positivity rates are observed in the 30–39 (48.1%) and 50–59 (44.4%) age groups, indicating that individuals within these ranges are more likely to test positive for LTBI. Conversely, younger adults (20–29) and older individuals (70–79 and 80–89) show predominantly negative results, with the oldest age groups exclusively yielding negative outcomes. This age-based distribution of positivity rates may suggest variations in exposure risk or immune response to LTBI across different life stages. However, the calculated correlation between age and LTBI positivity is approximately 0.013, which is nearly zero. This lack of a meaningful correlation suggests that age is not a strong predictor of LTBI positivity in this sample, implying that other factors beyond age may drive variations in LTBI positivity rates.

**Figure 3:**
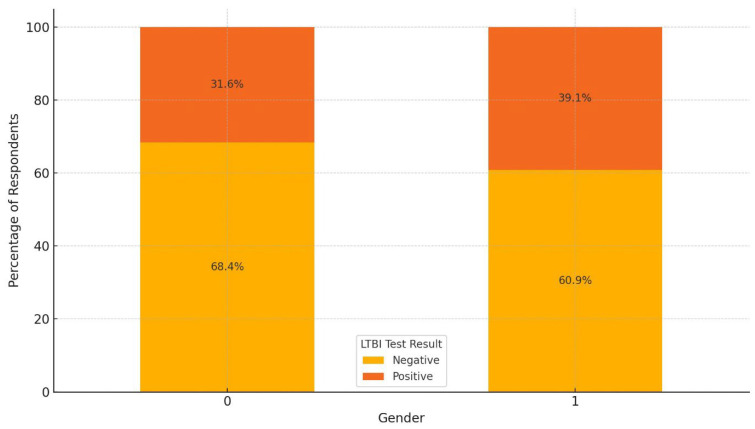
LTBI test results by gender.

In Figure 3, the LTBI test results reveal differences in positivity rates across gender categories. In females (0), 68.4% of respondents tested negative for LTBI, while 31.6% tested positive. In males (1), 60.9% tested negative, and 39.1% tested positive. This indicates that the female category has a higher percentage of negative results (68.4%) compared to those in the male category (60.9%). Conversely, the positivity rate is higher in males (39.1%) than in females (31.6%), suggesting that males may be more likely to test positive for LTBI. This observed disparity hints at a potential gender-related difference in LTBI prevalence within this sample.

**Figure 4:**
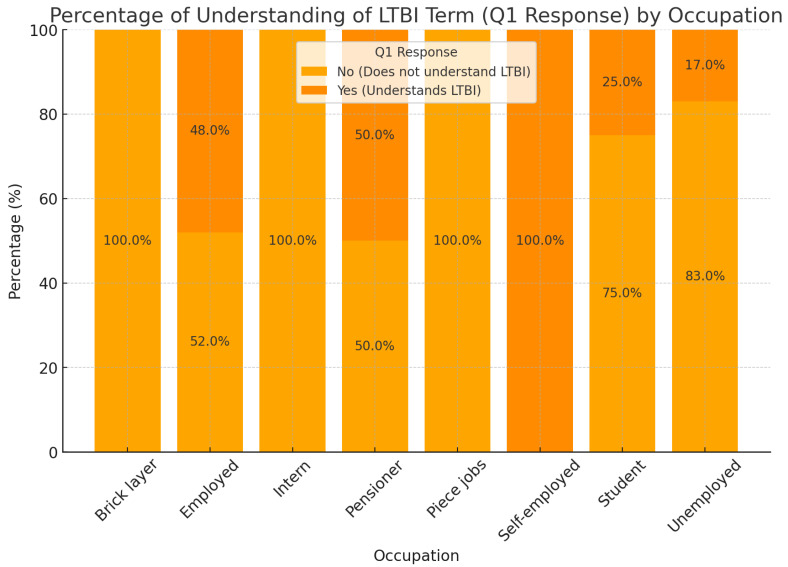
LTBI test results by occupation.

Figure 4 compares LTBI test results by occupation, providing insight into the relationship between different job categories and LTBI outcomes. The unemployed category is the largest and shows a substantial knowledge gap, with around 83% lacking an understanding of LTBI and only 17% demonstrating awareness. This points to a significant need for targeted education within this group. The employed category presents a more balanced understanding, with approximately 52% lacking and 48% possessing knowledge of LTBI, possibly due to greater access to health information within workplace environments. Smaller occupational groups, such as bricklayers, interns, and those in piece jobs, show a complete lack of understanding (100%), though they comprise a smaller portion of the sample. Meanwhile, the self-employed group, despite its small sample size, reports 100% understanding, while pensioners show minor levels of comprehension.

**Figure 5:**
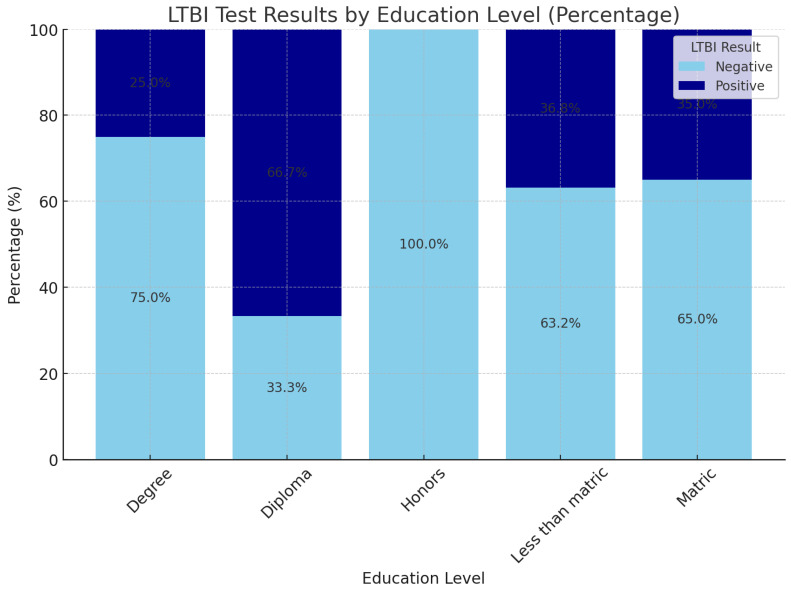
Q1 responses by education level.

Figure 5 compares Q1 responses across different education levels, providing insights into how educational background may influence participants’ views or knowledge related to the question posed in Q1. The analysis reveals that LTBI positivity rates vary by educational attainment, with higher rates among individuals with a Diploma (66.7%) and those with less than a Matric qualification (36.8%), suggesting that lower or vocational education levels may correlate with increased LTBI positivity. Individuals with Honors degrees show exclusively negative results in this sample, although this group has a small sample size, limiting generalizability. Degree holders and those with Matric qualifications present more balanced distributions, with positivity rates of 25% and 35%, respectively. These patterns indicate a potential trend where vocational diploma holders, at 66.7% positivity, represent the group with the highest LTBI positivity, followed by those with less than Matric and Matric qualifications, around 35–37% positivity. Degree holders show 25% positivity, while all Honors degree holders are negative for LTBI, though the latter group is smaller.

**Figure 6:**
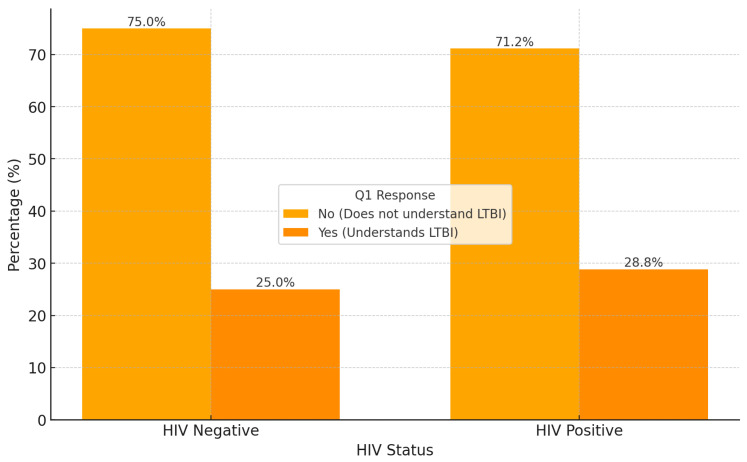
Q1 responses by HIV status.

Figure 6 illustrates the percentage of individuals’ understanding of the LTBI term, categorized by HIV status. In the HIV-negative group, 75.0% do not understand the LTBI term, while only 25.0% demonstrate an understanding, indicating a significant knowledge gap in LTBI awareness within this population. In comparison, within the HIV-positive group, 71.2% do not understand LTBI, and 28.8% show an understanding. Although the level of understanding is slightly higher among HIV-positive individuals compared to HIV-negative ones, a substantial portion in both groups still lacks adequate LTBI knowledge.

The logistic regression analysis for predicting LTBI test results based on demographic and survey factors achieved an accuracy of 70%. Precision metrics indicate that the model predicts negative results (77% precision) better than positive results (64% precision). Recall is relatively balanced between the classes, with 67% for negative and 75% for positive outcomes, leading to an F1-score of approximately 70% for both classes. Overall, the model performs reasonably well, demonstrating a balanced ability to identify both positive and negative LTBI statuses, though it shows a slightly stronger performance in predicting negative cases.

**Figure 7:**
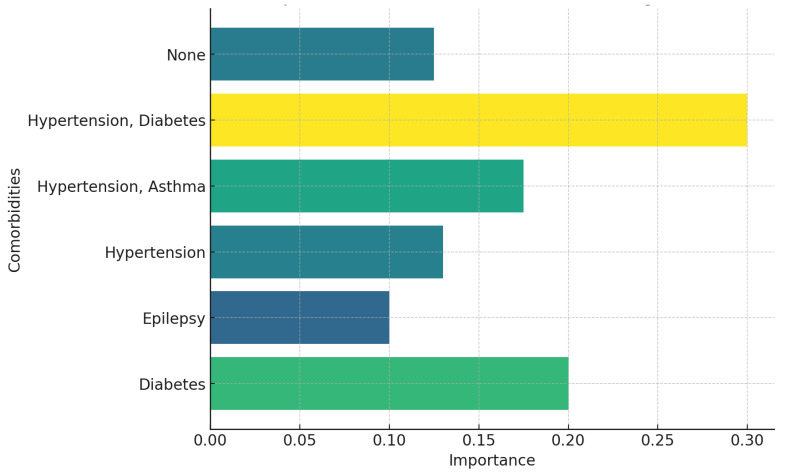
Feature importance for LTBI prediction to progression into active TB.

Patients with hypertension had ten LTBI-negative and five LTBI-positive results, which are the highest numbers of both positive and negative LTBI test results in this study. Patients with hypertension had a significant portion of those who tested positive. This suggests that hypertension may be a notable risk factor for LTBI positivity. There was one patient with both hypertension and asthma and one diabetic patient who tested positive for LTBI. Although the sample size is small, this suggests that comorbidities like diabetes and asthma combined with hypertension might be associated with an increased risk of LTBI positivity. Only one person with both diabetes and hypertension tested negative, which suggests that while these conditions are prevalent, they may not always coincide with LTBI positivity in this dataset. There is only one case of epilepsy with a negative result. This suggests that epilepsy alone may not have a strong association with LTBI positivity. The importance scores in Figure 7 highlight that individuals with both hypertension and diabetes (importance: 0.300) have the highest risk for TB progression among the comorbidity groups studied. This elevated risk likely stems from the compounded effects of these conditions on the immune system, increasing susceptibility to TB activation. Diabetes alone (importance: 0.200) also presents a significant risk factor, underscoring its role in TB progression due to its impact on immune function, which can facilitate the activation of latent TB infections. The combination of hypertension and asthma (importance: 0.175) shows a moderately high risk, indicating that these two conditions together may weaken respiratory health and immune response, further elevating TB susceptibility. Hypertension alone (importance: 0.130) carries a moderate importance score, suggesting it is relevant but less dominant than diabetes or its combination with other conditions; while hypertension may not directly increase TB risk, it can contribute indirectly through related health complications. Individuals with no comorbidities (importance: 0.125) have a relatively lower risk, which aligns with the expectation that, in the absence of additional health challenges, TB progression risk remains lower. Epilepsy (importance: 0.100) has the lowest importance score, suggesting it is the least associated with TB progression among these comorbidities. This lower score implies that while epilepsy may not directly increase TB risk, it could interact with other factors in specific populations, potentially contributing to a minor risk.

**Figure 8:**
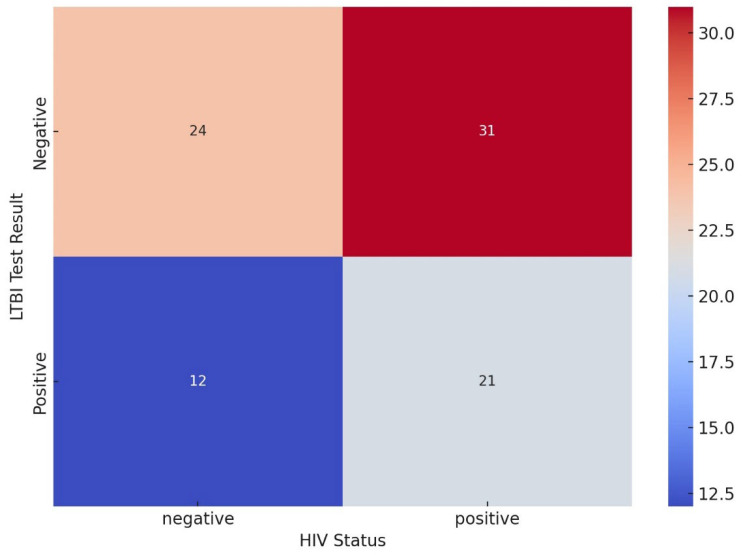
Correlation between LTBI test and HIV status.

The heatmap compares LTBI test results and HIV status and offers insights into the relationship between these two health outcomes. The top left (24) represents patients who are HIV-negative and had a negative LTBI test. The top right (31) represents individuals who are HIV-positive and had a negative LTBI test. The bottom left (12) represents individuals who are HIV-negative and had a positive LTBI test. The bottom right (21) represents individuals who are HIV-positive and had a positive LTBI test. These results suggest a relationship between HIV status and LTBI test results. There are slightly more individuals with a negative LTBI result among HIV-positive people compared to HIV-negative ones (Figure 8).

**Figure 9:**
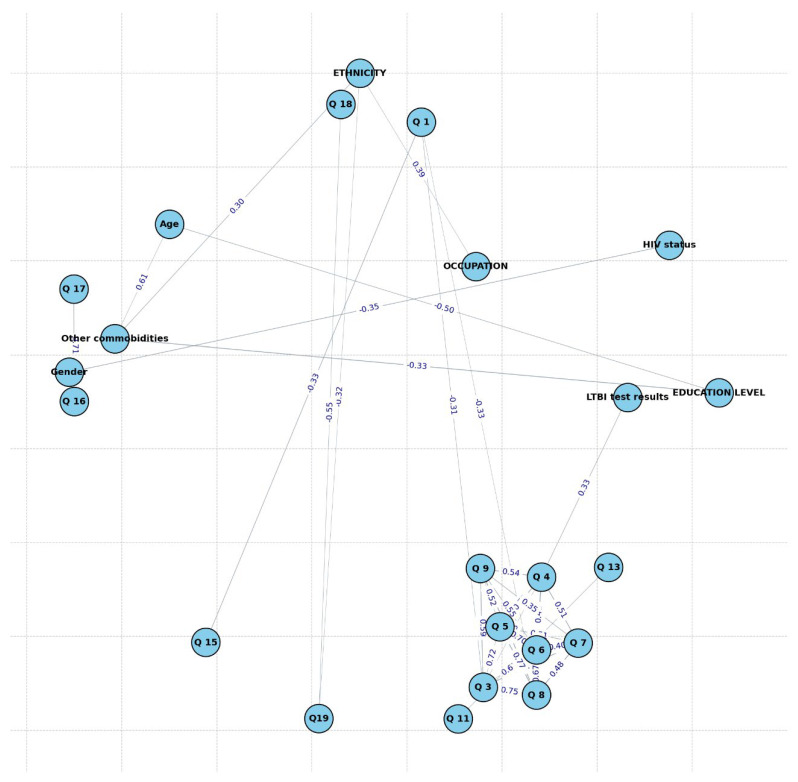
Network of significant variable correlations.

In Figure 9, the network analysis reveals important structural connections among variables, with nodes representing variables and edges (connections) indicating significant correlations (|correlation| > 0.3). The analysis reveals several significant correlations between demographic and survey variables within the LTBI study sample. There is a strong positive correlation of 0.50 between occupation and education Level, indicating that specific education levels may align with particular occupations, likely reflecting socioeconomic or career-related trends. Occupation also has a correlation of 0.35 with ethnicity, suggesting occupational roles vary across ethnic groups, potentially due to occupational segregation or cultural differences in employment trends. Gender and HIV status are correlated at 0.33, implying gender differences in HIV prevalence or diagnosis, possibly reflecting health disparities or risk factors by gender. Gender and education level have a negative correlation of −0.33, suggesting gender-based differences in educational attainment, where higher education levels may be less prevalent in one gender. Age correlates with education level at 0.32, indicating that educational attainment varies across age groups, with older individuals possibly having different levels of education than younger ones. Age is also correlated with other comorbidities at 0.61, highlighting that older individuals tend to have a higher prevalence of comorbid conditions, as is common with age-related health risks. Ethnicity and comorbidities have a negative correlation of −0.35, suggesting that certain ethnic groups may have different rates of comorbidities, with some groups potentially experiencing lower comorbidity burdens. Education level shows a correlation of −0.33 with LTBI test results, indicating that higher education might be associated with different risk or awareness levels related to LTBI. Among survey questions, Q8 and Q7 display a strong positive correlation of 0.75, suggesting these responses are closely related, likely reflecting similar constructs related to LTBI behaviors, knowledge, or attitudes. Similarly, Q9 and Q4 have a correlation of 0.54, implying that these items may capture related perceptions or knowledge that influence LTBI risk. The visualization of these interrelationships demonstrates the complex interplay of social, demographic, and health factors in shaping LTBI risk. These findings support the need for a holistic approach to LTBI management, integrating targeted health education, socioeconomic support, and tailored screening programs to address disparities and reduce the prevalence of LTBI.

The logistic regression model developed to predict LTBI test results shows moderate performance, achieving an overall accuracy of 67%. The precision for predicting negative cases (71%) is slightly higher than for positive cases (62%), indicating the model is somewhat better at identifying individuals without LTBI. Both positive and negative cases have a balanced recall rate of 67%, suggesting that the model captures each type of outcome with comparable effectiveness. The F1-scores are 0.69 for negative cases and 0.64 for positive cases, reflecting moderate predictive consistency across both classes. Overall, while the model demonstrates reasonable accuracy and balanced recall, its slightly higher precision in predicting negatives highlights an area for improvement in identifying positive LTBI cases.

**Figure 10:**
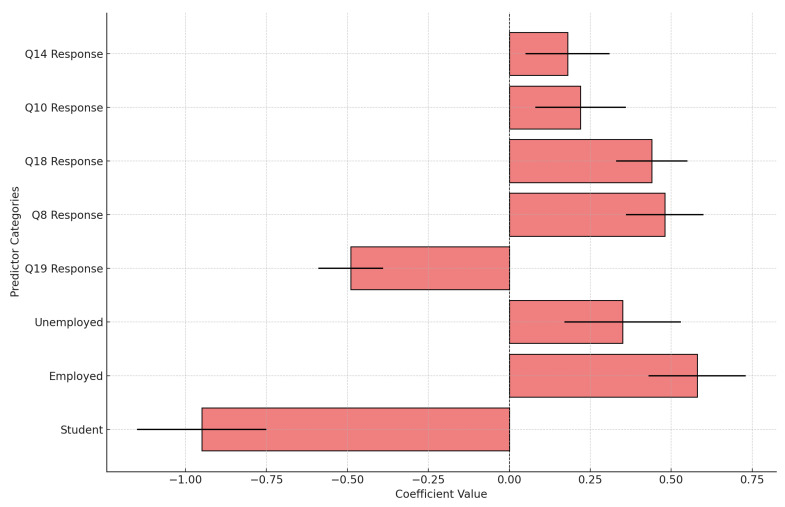
Various predictors of LTBI positivity on both negative and positive associations.

Figure 10 illustrates the impact of various predictors on LTBI positivity, highlighting both negative and positive associations. Among the negative predictors, being a student (−0.95) shows the strongest reduction in LTBI positivity likelihood, likely due to lower exposure risks in the student environment. Similarly, specific responses to Q19 (−0.49) are associated with decreased LTBI positivity, potentially reflecting protective behaviors or heightened awareness. On the other hand, positive predictors include being employed (+0.58), which increases the likelihood of LTBI positivity, possibly due to workplace exposure risks. Responses to Q 8 (+0.48) and 18 (+0.44) also show positive associations, suggesting that certain behaviors, knowledge gaps, or contexts contribute to increased LTBI risk. Q 14 responses (+0.18) have a weaker but still positive effect, indicating a more subtle influence on LTBI positivity. Larger coefficients, such as those for the student and employed categories, represent more pronounced impacts, while smaller coefficients, like Q14 response, reflect subtler effects. The precision of these estimates, as indicated by the error bars, varies, with narrower bars (e.g., Q19 response) suggesting higher confidence in the estimates compared to broader ones. This analysis underscores the diverse factors influencing LTBI positivity and their varying degrees of significance and reliability.

## 4. Discussion

The participants in our study were predominantly adults from various age groups, and we observed variability in LTBI prevalence based on factors such as gender, HIV status, and the presence of comorbidities. These demographic insights highlight how variables like occupation, education level, and health conditions affect LTBI positivity rates in our sample. For instance, higher positivity rates were observed in individuals with comorbidities such as hypertension and diabetes, which are prevalent in our sample and likely reflect the broader health landscape of rural South Africa. Similarly, there were differences in knowledge levels about LTBI, influenced by factors such as educational attainment and employment status, emphasizing the need for tailored health education.

The identification of LTBI is crucial in controlling TB transmission, as individuals with LTBI are at risk of developing active TB. Understanding the demographic factors influencing LTBI-negative results can inform public health strategies to reduce TB incidence.

The analysis of survey correlations on LTBI knowledge and attitudes aligns with findings in existing literature, illustrating common themes in public health understanding and knowledge gaps. The observed high positive correlations, particularly between questions related to understanding LTBI and its differences from active TB, reflect a broader pattern seen in other studies where knowledge of disease definitions is often interconnected with understanding disease progression and distinctions. For example, ref. [30] found that individuals who understand specific disease terminology are generally more informed about disease implications, suggesting that foundational knowledge in infectious diseases often correlates with more nuanced comprehension of related concepts [30].

Similarly, the strong correlation between questions on recommended treatments for LTBI and preventive measures reflects an established theme in health literacy research. According to [31], health literacy around treatment often overlaps with knowledge of preventive practices, particularly in infectious disease contexts. Their study demonstrated that individuals who are well-informed about treatment options are more likely to understand preventive strategies, indicating that health education interventions targeting both treatment and prevention could be jointly beneficial [31]. This synergy between treatment knowledge and preventive measures observed in the LTBI survey underscores the importance of an integrated approach to health education.

The notable negative correlations in this LTBI survey suggest knowledge gaps or contrasting perspectives, particularly among individuals with and without prior health education on TB. This divergence aligns with findings by [32], who noted that individuals without prior health education on tuberculosis often hold different perceptions about risk factors compared to those who are educated. Specifically, Odone et al. observed that prior health education significantly impacts risk perception, often leading to more cautious or accurate views on disease progression and risk factors [32]. This effect is echoed in the negative correlation between questions on health education and LTBI risk factors in the present survey, highlighting the role of education in shaping health perspectives. Other information indicates that individuals with higher levels of education tend to have a better understanding of LTBI and its associated risks; [33] found that improved education correlates with a reduction in LTBI risk as individuals become more aware of health risks linked to lifestyle choices and environmental factors such as poverty and overcrowding [33]. Similarly, Gao et al. highlighted that many respondents in their study had low TB knowledge, often confusing LTBI with active TB disease, which can lead to misconceptions about the necessity of treatment [34]. This confusion underscores the need for targeted educational materials that accurately convey information about LTBI, its transmission, and treatment options [34].

Furthermore, the moderate negative correlation between awareness of LTBI progression to active TB and general understanding of LTBI could reflect a broader trend identified in public health studies. According to [35], awareness of disease progression is sometimes inversely related to specific disease knowledge among the general public. This suggests that individuals may recognize certain risks or progressions without fully grasping underlying terms or definitions, which is consistent with the current survey findings on LTBI [35].

The weaker negative correlation between perceptions of LTBI as a public health concern and understanding of LTBI itself may indicate a subtle gap between general awareness and specific knowledge, which has been observed in other areas of health research. For instance, ref. [36] found that general concerns about health issues do not always correlate strongly with specific knowledge or understanding, especially when the issue involves complex medical terminology [36]. This pattern suggests that raising general awareness of LTBI as a health concern may not necessarily translate into an improved understanding of the disease, pointing to the need for targeted education strategies. These correlation patterns within the LTBI survey align with established findings in public health literature, underscoring the relationship between foundational knowledge and thematic understanding, the impact of prior health education on perceptions, and the occasional disconnect between general awareness and specific understanding [32,33,34,35]. Addressing these thematic clusters and knowledge gaps could guide future educational interventions to improve a comprehensive understanding of LTBI.

In LTBI-negative patients, certain age groups, such as older or younger groups, showed a higher proportion of LTBI-negative results, indicating lower risk or exposure to TB, while younger age groups (e.g., 20–29, 30–39) had more LTBI-negative results which may suggest that these patients have better access to preventive healthcare or live in less risky environments. Older adults, particularly those over 65 years, often show a higher proportion of LTBI-negative results, which may reflect a combination of factors, including decreased exposure to TB due to improved living conditions and healthcare access over time [37]. We recognize that the small sample size within the 70–79 age group could impact the robustness of findings, as smaller samples can result in reduced statistical power and a less representative dataset. Even though this was a pilot study with a limited overall sample (n = 88), this age group showed exclusively negative LTBI results; our calculated correlation between age and LTBI positivity was near zero, suggesting no significant relationship between age and LTBI positivity within our sample.

Socioeconomic factors play a role in younger patients, which is a benefit from improved living conditions and education about TB prevention [38]. The age groups of LTBI-positive patients with a higher proportion of LTBI-positive results may indicate that older individuals or those within specific age ranges are more vulnerable to TB [38]. A higher count of LTBI-positive results in older groups could suggest accumulated exposure over time or reduced immunity as individuals age. Older adults often have a longer exposure history to TB, which can lead to a higher likelihood of LTBI positivity. Several studies have documented that individuals who have lived in areas with high TB prevalence or have had close contact with infectious TB cases are at increased risk of developing LTBI [37]. Historically, older adults may have been exposed to TB before the advent of effective public health measures, such as vaccination and improved sanitation. This accumulated exposure can result in a higher prevalence of LTBI in this demographic [39].

There is an age-specific risk observed in our study, as individuals age, their immune systems undergo changes that can affect their ability to control latent infections. The decline in immune function, often referred to as immunosenescence, can lead to an increased risk of LTBI positivity and progression to active TB disease. Research indicates that older adults may have a diminished immune response to *Mtb*, making it more challenging for their bodies to contain the infection. This reduced immune efficacy can result in a higher likelihood of LTBI positivity [40]. Older adults often present with comorbidities that can further compromise immune function, such as diabetes, chronic lung disease, and malignancies. These conditions can increase susceptibility to TB infection and may contribute to the higher rates of LTBI positivity observed in older populations [41]. The analysis reveals a clear trend in LTBI positivity rates across age groups. Younger individuals (20–29 years) exhibit the lowest positivity rates, while older age groups (30–39 years and above) show increasing positivity rates. The findings reflect various factors, including historical exposure to TB, changes in public health policies, and variations in age-related immune response [42]. The absence of positive results in the 70–79 age group warrants further investigation. It may indicate effective public health interventions over the years or a demographic shift in exposure patterns [43]. Focused screening and preventive measures should be considered for age groups with higher positivity rates, particularly those aged 30–59.

Previous studies have shown that populations with limited access to health education often have lower awareness of LTBI, which can lead to higher rates of progression to active TB [1,44]. In a related context, a systematic review indicated that LTBI prevalence rates can be particularly high in vulnerable populations, such as refugees and asylum seekers, where rates were reported as high as 23% [45]. This aligns with findings from other studies in similar settings, suggesting that rural populations in South Africa may exhibit comparable or higher prevalence rates due to socioeconomic factors and healthcare access issues [44,46].

The findings from this pilot study highlight the urgent need for improved education and awareness regarding LTBI among patients in rural Eastern Cape. The lack of knowledge about LTBI can hinder early detection and treatment, increasing the risk of progression to active TB, which remains a leading cause of morbidity and mortality in South Africa [1,46]. Moreover, the high prevalence of LTBI reported in this study is consistent with global trends, where populations characterized by socioeconomic challenges often show elevated rates of LTBI [47]. For instance, studies have documented LTBI prevalence rates among healthcare workers and other high-risk groups, emphasizing the need for focused screening and preventive measures [48].

The distribution of LTBI-negative and LTBI-positive patients by occupation provides insight into the potential risk factors for TB exposure. Occupations with a higher proportion of LTBI-negative patients suggest lower TB exposure risks, potentially due to better job conditions, access to healthcare, or preventive measures [49]. Conversely, occupations with higher LTBI-positive rates, such as unemployment, indicated increased vulnerability due to factors like poor living conditions, limited healthcare access, or working in environments with higher TB exposure risks [50]. These findings highlight the importance of occupational and socioeconomic factors in TB prevention

Education is crucial in shaping individuals’ knowledge and awareness of health issues, including LTBI. Our results indicate that patients with higher education levels tend to have better health literacy, which is essential for understanding complex health issues such as LTBI. Higher education often correlates with increased access to information and resources, enabling individuals to make informed health decisions [51]. Responses to Q1 by education level suggest that patients with higher education (e.g., matric or beyond) prefer certain responses, indicating that education could influence knowledge or awareness of LTBI knowledge. Lower education levels exhibit different response patterns than higher education levels, highlighting gaps in understanding or awareness based on education [52]. Our findings reveal that educational attainment shapes perspectives on the issue. Higher education may correlate with more informed or specific responses, while lower education levels could indicate a need for more targeted information or awareness campaigns. A study by [51] indicated that educated individuals were more aware of the risks associated with untreated LTBI, leading to a higher likelihood of seeking treatment.

Hypertension emerges as the most critical factor in predicting LTBI positivity, indicating it is a substantial predictor for LTBI, especially among female patients. This aligns with evidence that hypertension may impair immune function, increasing susceptibility to infections, including LTBI [51]. The absence of comorbidities ranks as the second most influential factor, suggesting that even without additional health burdens, individuals’ general health profile may impact LTBI results. When hypertension is combined with diabetes, it becomes an even stronger predictor of LTBI positivity than either condition alone, underscoring the compounded impact of these conditions on infection risk [52]. While diabetes alone is somewhat less significant than hypertension or combined comorbidities, it still plays a role in LTBI risk, hinting that diabetes may be a moderate risk factor. The combination of hypertension and asthma also contributes to LTBI prediction, albeit to a lesser extent, indicating that asthma may not significantly enhance LTBI risk when coupled with hypertension.

In contrast, epilepsy and the combination of diabetes with hypertension appear to have minimal or no predictive impact on LTBI positivity, suggesting they are weaker indicators of risk. The low predictive score for individuals without comorbidities aligns with expectations that individuals without additional health challenges generally have lower TB progression risks. However, it is important to note that TB can still affect healthy individuals, particularly in high-exposure environments, highlighting the need for broad community screening and preventive measures despite lower individual risk in those without comorbidities.

Epilepsy shows the lowest importance score among examined conditions, implying it has the least association with TB progression. Although epilepsy itself may not directly contribute to TB risk, it could impact disease progression in specific contexts, potentially due to interactions with anti-epileptic medications or other health complications. This finding suggests epilepsy should not be a primary focus in TB screening efforts but may still warrant attention in comprehensive health assessments where other risk factors are present.

This study indicates that hypertension, especially in conjunction with other chronic conditions like asthma or diabetes, plays a substantial role in LTBI risk. This suggests that individuals with hypertension, particularly those with additional comorbidities, should be closely monitored for LTBI. The combined effects of hypertension, diabetes, and other conditions may create an environment where the immune system is less effective at controlling latent infections, increasing the likelihood of LTBI progressing to active TB. These findings highlight the importance of assessing multiple comorbidities when evaluating LTBI risk, as individuals with multiple chronic conditions face a heightened risk due to cumulative impacts on immune function and overall health [52].

The combination of LTBI-negative and HIV-negative had a high count, suggesting that a significant portion of the patients are free from both LTBI and HIV, which may reflect a lower overall health risk in this group. A notable number of LTBI-positive and HIV-negative might patients indicates that patients who are free from HIV could still be susceptible to LTBI, suggesting that the two conditions do not necessarily occur together. The coexistence of LTBI and HIV is a well-documented phenomenon, with HIV significantly increasing the risk of progression from LTBI to active TB [53]. However, a notable number of LTBI-positive patients may be HIV-negative, indicating that individuals without HIV can still be susceptible to LTBI. Several factors contribute to LTBI susceptibility in HIV-negative individuals, including socioeconomic status, living conditions, and occupational exposure. Individuals living in crowded or poorly ventilated environments are at increased risk of TB exposure, regardless of their HIV status [52]. Additionally, certain occupations, such as healthcare work, can increase the likelihood of LTBI positivity among HIV-negative individuals [52]. The combination of LTBI-negative and HIV-positive had a significant count, suggesting that patients with HIV may not always have LTBI, indicating that effective treatments or interventions might be limiting co-infections in this group. A higher number of LTBI-positive and HIV-positive points to a potential correlation between LTBI and HIV, where individuals with HIV may be at increased risk of developing LTBI due to weakened immune systems or overlapping risk factors [54]. Our study reveals that co-occurrences of LTBI-positive and HIV-positive group patients with HIV are more prone to LTBI infections. The correlation between HIV and LTBI is marked by significant immune response interactions, clinical management complexities, and critical treatment strategy implications. HIV infection compromises immune function primarily by depleting CD4+ T-cell counts, which are pivotal in controlling Mycobacterium tuberculosis in latent infections. This immune suppression heightens susceptibility in HIV-infected individuals to LTBI reactivation, underlining the importance of CD4+ cells in TB immunity [51]. Managing HIV in patients with LTBI is essential as antiretroviral therapy (ART) can partially restore immune function and decrease TB incidence. However, ongoing LTBI surveillance remains crucial for HIV-positive patients because immune compromise often persists even with ART. Integrated care is required to manage LTBI and HIV effectively; preventive treatments such as isoniazid preventive therapy are recommended for HIV-positive individuals, especially in high-TB-burden regions, to mitigate the progression to active TB. Such dual-focused management is crucial for TB control, significantly impacting the morbidity and mortality associated with TB in HIV-positive populations and supporting global TB elimination goals. Some studies found that patients with HIV are at an increased risk of LTBI due to several factors, including immune system compromise and shared risk factors for both infections [51]. The notable health risk patterns in our study reveal that lower numbers in LTBI-positive groups for HIV-negative patients could indicate that LTBI is more closely related to specific vulnerabilities or conditions like HIV. The notable presence of LTBI-positive patients who are HIV-negative indicates that susceptibility to LTBI is not limited to patients with HIV.

The network analysis of correlations among demographic and survey variables in the LTBI study sample provides valuable insights into the interrelationships within this population. Significant correlations (|correlation| > 0.3) suggest structural patterns and highlight sociodemographic and health-related trends that align with findings in public health literature.

One prominent finding is the positive correlation between occupation and education level (0.50), indicating that higher educational attainment is associated with specific occupations. This relationship aligns with findings from [55], which suggest that education is often a predictor of occupation due to socioeconomic factors and career progression pathways that require higher qualifications [55]. The observed correlation between occupation and ethnicity (0.35) may reflect occupational segmentation based on ethnic backgrounds, potentially due to sociocultural influences or disparities in employment opportunities, which have been documented in studies such as Jones et al. (2017) [56].

The correlation between gender and HIV status (0.33) suggests gender disparities in HIV prevalence or diagnosis, potentially due to risk factors or health access issues. This pattern is consistent with research indicating that HIV infection rates often differ by gender, influenced by biological susceptibility and social determinants of health [57]. Additionally, the negative correlation between gender and education level (−0.33) indicates disparities in educational attainment between genders, which aligns with global trends in gender-based educational inequalities, particularly in resource-limited settings [58].

Age shows notable associations with health-related variables. Its correlation with other comorbidities (0.61) underscores the increased risk of comorbid conditions with aging, a well-documented phenomenon linked to age-related health deterioration and chronic disease prevalence [59]. The correlation between age and education level (0.32) reflects that educational attainment varies across age groups, possibly due to historical changes in educational access or socioeconomic factors affecting older generations differently, a trend highlighted in studies on education and aging populations [60].

The negative correlation between ethnicity and comorbidities (−0.35) may suggest disparities in comorbidity prevalence among ethnic groups, potentially due to differences in genetic predispositions, lifestyle factors, or healthcare access. This finding resonates with research on health disparities across ethnic groups, as documented by [61], underscoring social determinants’ impact on health outcomes [61]. Additionally, education level negatively correlates with LTBI test results (−0.33), implying that higher educational levels might correlate with reduced LTBI positivity rates. This aligns with findings that education often enhances health literacy and access to preventive care, reducing infectious disease prevalence [62].

Within the survey questions, Q8 and Q7 exhibit a strong positive correlation (0.75), suggesting that responses to these questions are closely related, potentially reflecting similar attitudes or knowledge regarding LTBI preventive behaviors or risks. This pattern of related survey responses is often observed in knowledge and behavior assessments, where similar constructs yield correlated responses [63]. Similarly, the correlation between Q9 and Q4 (0.54) implies that these questions capture related perceptions or knowledge aspects, possibly influencing respondents’ awareness or attitudes toward LTBI.

Overall, this network analysis reveals significant correlations that reflect both demographic trends and knowledge patterns within the LTBI study sample. These findings highlight areas where socioeconomic and demographic factors may impact health outcomes and attitudes, providing insights into the potential focus areas for targeted health education and interventions.

The logistic regression analysis reveals several key factors associated with LTBI positivity, highlighting the diverse demographic and behavioral influences on infection risk. The analysis underscores occupation as a significant predictor, with occupation_student showing a large negative coefficient (−0.95), suggesting that students are less likely to test positive for LTBI. This may be due to reduced exposure to high-risk environments, as students typically spend more time in controlled academic settings rather than in workplaces where exposure risk is elevated. Conversely, occupation_employed has a positive coefficient (+0.58), indicating a higher likelihood of LTBI positivity among employed individuals, potentially due to workplace-related exposure risks or socioeconomic factors linked to employment, particularly in high-risk sectors such as healthcare and manual labor [64,65].

Educational attainment also appears influential, with education level_diploma associated with an increased likelihood of LTBI positivity. This may reflect socioeconomic backgrounds or occupational patterns associated with this educational level, exposing individuals to higher LTBI risk. This finding aligns with previous research showing that education indirectly impacts TB risk through factors like employment type and access to healthcare [66].

Survey responses further shape LTBI positivity predictions. Q19 has a negative coefficient (−0.49), suggesting that certain responses are linked to reduced LTBI positivity, potentially reflecting preventive behaviors or heightened awareness of LTBI risks. This corresponds with studies showing that awareness and preventive actions mitigate TB risk in high-exposure populations [67]. Conversely, Q8 (+0.48) and Q18 (+0.44) show positive coefficients, implying these responses may correspond to behaviors, attitudes, or knowledge gaps associated with higher LTBI risk, potentially due to limited understanding of preventive practices [68].

Other factors also contribute to LTBI prediction, though to a lesser extent. For example, occupation_unemployed has a modest effect, suggesting that while unemployment may limit exposure, it may correlate with socioeconomic vulnerabilities that impact health status and TB risk [69]. Additionally, positive coefficients for Q10 and Q14 suggest that specific responses to these questions may indicate behaviors or attitudes that elevate infection risk, highlighting areas where targeted educational interventions could help reduce LTBI prevalence. This multifaceted risk profile points to occupation, education level, and awareness as critical factors in LTBI management and intervention design.

In analyzing LTBI understanding by occupation, distinct patterns emerge across different occupational groups. The notably higher understanding among employed individuals suggesting employment may enhance access to health education. Conversely, the low understanding within the unemployed and informal occupation groups signals a potential benefit from targeted LTBI awareness and educational initiatives focused on these vulnerable populations. The overall high proportion of individuals lacking understanding highlights a need for increased education on LTBI across both HIV statuses, with a marginally higher awareness observed in the HIV-positive group (28.8% vs. 25.0%). These correlations provide insights into the interrelationships among demographic and health-related factors in this sample, identifying clusters that may inform targeted interventions or further research. Gender shows notable connections with both HIV status and education level, suggesting that gender differences may influence health outcomes and educational backgrounds in this sample. Education level is linked to both gender and age, indicating an expected pattern where educational attainment varies with age groups. Occupation strongly correlates with ethnicity and education level, implying that occupational distribution may differ by ethnic background and education, potentially reflecting socioeconomic trends within the sample. Age correlates with both comorbidities and education level, suggesting that older individuals may have varied educational backgrounds and a higher prevalence of comorbid conditions. Ethnicity is significantly associated with occupation and comorbidities, which may highlight disparities in health outcomes and occupational distributions across ethnic groups. This network graph highlights clusters of related variables, offering insights into demographic patterns within the sample. Occupation and education level emerge as central variables, connecting with ethnicity, gender, and age, while health-related factors (such as comorbidities and HIV status) are linked with demographic characteristics, suggesting areas for targeted health interventions.

The high prevalence of LTBI observed in our study setting emphasizes the need for targeted public health interventions within rural South African communities. Our study identifies specific LTBI risk factors, including comorbidities such as hypertension and diabetes and socioeconomic factors like unemployment, that suggest local health policies should prioritize LTBI screening and prevention within primary healthcare settings. Such policies could encompass training healthcare workers to recognize high-risk individuals, facilitating early diagnosis and preventive treatment to reduce LTBI progression to active TB. On a national scale, these findings underscore the importance of integrating LTBI management into South Africa’s TB control initiatives, with a focus on resource allocation to high-prevalence, socioeconomically challenged areas. National policy efforts could also expand public health education, particularly within populations with limited healthcare access, to reduce misconceptions about LTBI and increase preventive measure adherence, supporting national TB elimination goals. Internationally, this study contributes to the global understanding of LTBI in high-burden areas, in line with the World Health Organization’s End TB Strategy. The associations between LTBI and comorbidities and occupational factors point to the need for standardized global guidelines that target LTBI screening and intervention in high-risk populations. Additionally, our findings support global advocacy for sustained funding and resources, especially for rural and underserved regions where TB prevalence remains high. Collectively, these findings advocate for multilayered policies that strengthen LTBI screening, education, and prevention across local, national, and international levels, advancing more effective TB control in rural and socioeconomically disadvantaged communities. There is a need for follow-up studies with broader geographic and demographic coverage to enhance the external validity of the results. Despite the small sample size, the study provides valuable insights into LTBI risk factors and knowledge gaps in a high-burden region, which can inform strategic interventions and public health policies.

## 5. Conclusions

This pilot study emphasizes the substantial burden of LTBI among a rural clinic population in the Eastern Cape, South Africa, and highlights the importance of early diagnosis and public health education in mitigating TB transmission. The correlation analysis suggests that patients have clustered knowledge in certain areas but reveals gaps between general awareness and specific, actionable understanding. Enhancing both foundational knowledge and providing detailed, practical information could bridge this gap, fostering effective health behaviors. Findings indicate that LTBI prevalence varies across demographics, with younger individuals showing lower positivity rates, likely due to better access to preventive healthcare. Conversely, older adults and those with comorbidities, such as hypertension, asthma, and diabetes, particularly among female patients, exhibit higher LTBI positivity, suggesting that conditions like hypertension may be key predictors of LTBI risk and warranting targeted monitoring and preventive interventions for these at-risk groups.

Additionally, the study identifies significant knowledge gaps about LTBI among individuals with lower educational attainment, underscoring the need for targeted public health efforts to enhance LTBI awareness in rural and underserved communities. Bridging these gaps is essential for empowering communities to seek timely care and treatment, ultimately reducing the progression from LTBI to active TB. This study provides valuable insights into LTBI epidemiology in a high-TB-burden area, reinforcing the importance of integrating LTBI screening and education into routine healthcare services. Larger studies are required to confirm these findings and inform focused interventions to reduce TB incidence and mortality in rural South Africa. While the specific regional focus may limit broader applicability, this study raises critical public health concerns relevant to similar high-burden, resource-limited areas. Addressing these issues could yield valuable insights into LTBI dynamics in underserved regions, often underrepresented in TB research. Although the study’s specific regional focus may limit the broader generalizability of the findings, it underscores critical public health concerns for similar high-burden, resource-limited settings. Addressing these challenges can contribute valuable insights into LTBI dynamics in underserved areas often underrepresented in TB research. Future studies could include broader geographic and socioeconomic groups to enhance the diversity of the sample and further strengthen generalizability.

## Figures and Tables

**Table 1:** Knowledge of LTBI survey questions.

Question Code	Question
Q1	Have you ever heard of latent tuberculosis infection (LTBI) before?
Q2	Have you ever received health education on LTBI and TB?
Q3	What do you understand by the term “latent tuberculosis infection”?
Q4	How is LTBI different from active tuberculosis (TB)?
Q5	What are the risk factors for developing LTBI?
Q6	What are the possible consequences of having untreated LTBI?
Q7	Can LTBI progress to active TB?
Q8	What are the recommended treatments for LTBI?
Q9	Are there any preventive measures individuals with LTBI should take to avoid developing active TB?
Q10	Do you think LTBI is a significant public health concern?
Q11	How concerned are you about the possibility of progressing from LTBI to active TB?
Q12	Do you believe that LTBI treatment is necessary, even if you do not have symptoms?
Q13	How do you perceive the importance of LTBI screening programs?
Q14	What barriers do you think may prevent individuals from seeking LTBI testing or treatment?
Q15	Have you ever been screened for LTBI?
Q16	If you tested for LTBI, did you receive treatment?
Q17	If you received treatment for LTBI, did you complete the entire course of medication?
Q18	Have you ever been in close contact with someone diagnosed with active TB?
Q19	If yes, did you seek medical evaluation or testing for LTBI?

## Data Availability

Data can be requested from the corresponding author.
